# A novel Bayesian geospatial method for estimating tuberculosis incidence reveals many missed TB cases in Ethiopia

**DOI:** 10.1186/s12879-017-2759-0

**Published:** 2017-10-02

**Authors:** Debebe Shaweno, James M. Trauer, Justin T. Denholm, Emma S. McBryde

**Affiliations:** 1 Department of Medicine, University of Melbourne, Melbourne, VIC Australia; 2 School of Public Health and Preventive Medicine, Monash University, Melbourne, Australia; 3 Victorian Tuberculosis Program at the Peter Doherty Institute for Infection and Immunity, Melbourne, VIC Australia; 4 Department of Microbiology and Immunology, University of Melbourne, Melbourne, VIC Australia; 5 Australian Institute of Tropical Health & Medicine, James Cook University, Townsville, QLD Australia

**Keywords:** Tuberculosis, Incidence, Spatial analysis, Binomial mixture models

## Abstract

**Background:**

Reported tuberculosis (TB) incidence globally continues to be heavily influenced by expert opinion of case detection rates and ecological estimates of disease duration. Both approaches are recognised as having substantial variability and inaccuracy, leading to uncertainty in true TB incidence and other such derived statistics.

**Methods:**

We developed Bayesian binomial mixture geospatial models to estimate TB incidence and case detection rate (CDR) in Ethiopia. In these models the underlying true incidence was formulated as a partially observed Markovian process following a mixed Poisson distribution and the detected (observed) TB cases as a binomial distribution, conditional on CDR and true incidence. The models use notification data from multiple areas over several years and account for the existence of undetected TB cases and variability in true underlying incidence and CDR. Deviance information criteria (DIC) were used to select the best performing model.

**Results:**

A geospatial model was the best fitting approach. This model estimated that TB incidence in Sheka Zone increased from 198 (95% Credible Interval (CrI) 187, 233) per 100,000 population in 2010 to 232 (95% CrI 212, 253) per 100,000 population in 2014. The model revealed a wide discrepancy between the estimated incidence rate and notification rate, with the estimated incidence ranging from 1.4 (in 2014) to 1.7 (in 2010) times the notification rate (CDR of 71% and 60% respectively). Population density and TB incidence in neighbouring locations (spatial lag) predicted the underlying TB incidence, while health facility availability predicted higher CDR.

**Conclusion:**

Our model estimated trends in underlying TB incidence while accounting for undetected cases and revealed significant discrepancies between incidence and notification rates in rural Ethiopia. This approach provides an alternative approach to estimating incidence, entirely independent of the methods involved in current estimates and is feasible to perform from routinely collected surveillance data.

**Electronic supplementary material:**

The online version of this article (10.1186/s12879-017-2759-0) contains supplementary material, which is available to authorized users.

## Background

Population level tuberculosis (TB) prevalence and incidence studies are resource and time-intensive, and so impractical for regular evaluation of TB trends in most settings. Almost universally, data acquired from routine programmatic TB notifications are adjusted by a number of methods, including expert opinion regarding case detection rates (CDR) in the local context, to estimate and report TB incidence [1]. In the few countries with recent and well-conducted prevalence surveys, incidence is calculated from a combination of these survey findings and estimates of the duration of disease, with the latter derived from the pre-chemotherapy era or from mathematical models [2, 3]. Because of uncertainties in the duration of disease, incidence estimates from these methods differ and optimal methods remain elusive [2]. As a result, significant uncertainty exists regarding the true TB incidence and other programmatically important unobserved values, such as CDR [4].

Given these uncertainties, notification data with imputation of estimated missing cases have been relied on in many epidemiological TB studies, including in spatiotemporal characterization studies [5–8]. Failure to appropriately account for missed cases could reduce the power of such studies to detect factors affecting population-level TB dynamics, and introduce systematic bias of estimates of the effect of covariates towards the null [9]. Both factors could obscure important population-level patterns. Similarly, interpretation of maps from these studies is problematic, as spatial patterns in notification data could reflect spatial dynamics in the true underlying incidence or case detection performance. Thus these maps could be biased to areas with better case detection performance, leading to resource misallocation. One such cause of bias is systematic under-reporting which could also vary depending on the availability of health care facilities.

To address this bias, we aimed to estimate TB incidence and case detection in Ethiopia using an alternative Bayesian methods based on routinely collected surveillance data, without the need for expert opinion regarding programmatic performance or estimates of duration of disease.

## Methods

We collected data on TB patients diagnosed between 2010 and 2014 in all 66 *kebeles* (the smallest geographical administrative unit in Ethiopia) of Sheka Zone (a remote zone in the country). These TB cases were pooled based on *kebele* of residence and year. We produced population density using census data and *kebele* shape files obtained from the Central Statistical Agency (CSA). Data on health facility availability were obtained from the Zonal health department. At the time of data collection, close to 50% of health facilities in the Zone did not have sputum microscopy, and there was no access to chest x-ray and culture facilities. Further details of the data and the study area are presented elsewhere [10].

### Model development

Hidden Markov models (Bayesian geospatial binomial mixture models) were developed to estimate the true underlying but unknown TB incidence and case detection rates. Hidden Markov models(HMMs) are flexible time series models for sequences of observations that are known to be driven by an underlying state which is hidden from the observer yet is in some way predictable, such as being serially auto-correlated, geospatially correlated or through predictor variables that are observable. The value of HMMs is their ability to predict the underlying process; which they do provided the second process (the relationship between hidden and observed data) is one that itself follows rules that can be defined and in turn estimated.

Here, the underlying process is the factors that drive TB incidence in the study region. We have information including the population density, serial data and geospatial position of the *kebele*. The process that relates incidence to notification is the case detection rate or the proportion of cases that are notified. Given that (by definition) notification is the product of incidence and case detection rate, the natural model choice is the binomial relationship between the observation (notification) and hidden state (incidence). We also allow for *kebele*-specific effects on incidence, in effect leading to a (higher variance) beta-binomial distribution for observed notifications in each *kebele*.

Such a model when challenged by data can lead to issues of identifiability in that both high incidence/low case detection and low incidence/high case detection can explain the same notification rate. However, with sufficient information (e.g. predictors of incidence, changes over time and further information to assist in the observation model, such as presence of a health centre), the precision of model estimates can increase.

The models are informed by spatially and temporally replicated TB case counts and yield estimates of the true incidence and case detection. The parameters of this state process describe the spatiotemporal variation in incidence, which is considered as a latent variable by the model and is our key output of interest.

### The state-space (the true underlying incidence) model

The number of incident TB cases (the latent state) in site *i* and year *j,* conditional on the expected mean λ_ij_ is a realisation of a Poisson distribution, where the expected number λ_ij_ is a product of the per capita TB rate (π_ij_, measured as a probability between 0 and 1) and the susceptible population size in year *j* at site *i*:

1 $$ {Incidence}_{ij}\sim Poisson\ \left({\lambda}_{ij}\right) $$

2 $$ {\lambda}_{ij}={\pi}_{ij}\times {Population}_{ij}, $$

The site index *i* runs from 1 to 66, representing the 66 *kebeles* and the year index *j* runs from 1 to 5. The logit transformed probability of incident TB (*π*_*ij*_ *)* is, in turn, a logit-linear function of the site- and year-specific covariates where population density (*X*_*i*_), average incidence rate in *kebeles* that share a border with the index *kebele (Z*_*ij*_ *)*, and logit transformed incidence rate at a temporal lag of one year *(π*_*ij-1*_) with intercept *β*_*0*_ and slopes *β*_*1*_ *, β*_*2*_ and *β*_*3*_ (eq. 3) were fitted as fixed effects. Extra-Poisson dispersion in the incidence is accounted by specifying two types of random effects: a spatially correlated random effect (ɛ) and a non-spatially correlated random effect (ν).

3 $$ \log it\left({\pi}_{ij}\right)=\beta +{\beta}_1{X}_i+{\beta}_2{Z}_{ij}+{\beta}_3\log it\left({\pi}_{ij-1}\right)+{v}_{ij}+{\varepsilon}_i $$

Spatial dependence was introduced into regression in two ways: by introducing spatially structured random error and spatial lag. The spatially structured random effect, **ɛ** = (ɛ_1_, … ɛ_66_), accounts for the effect of spatial proximity, with the prior distribution taken as a Gaussian Conditional Autoregressive function (CAR), in which the prior probability distribution of the value of ɛ_i_ has a mean equal to the weighted average of the neighbouring random effects [11, 12] and variance following an inverse gamma distribution (shape = 0.5, scale = 0.0005). Average TB notification rate in the *kebeles* adjacent to an index *kebele* was used to define spatial lag.

### The case detection model

Notified TB cases have two sources of variation: variation in the true underlying incidence (*Incidence*_*ij*_) and variation in the case detection process (*P*_*ij*_ *,*). As a description of the detection process giving rise to detected (observed) cases, we assumed detected cases (*Y*_*ij*_) by health facilities are realisations of a binomial process conditional on the underlying true incidence and case detection probability. The logit transformed case detection probability (*P*_*ij*_) is a linear function of the covariate of health facility (*H*) availability.

4 $$ {Y}_{ij}\sim Binomial\left({P}_{ij},{Incidence}_{ij}\right), $$

5 $$ \log it\left({P}_{ij}\right)=\theta +{\theta}_1{H}_i+{\omega}_{ij} $$

Hence θ_1_ is the log(odds ratio) of detection in the presences of a health centre compared with no health centre. To account for additional heterogeneity in CDR not captured by this covariate, we fitted a normally distributed random effect that differed by *kebele* and year (*ɷ*_*ij*_). The prior distribution of this error term had a mean of zero and a standard deviation drawn from the uniform (0, 5) distribution.

A non-informative uniform prior distribution (−10, 10) was chosen for all regression coefficients and intercept parameters (other than those already specified) to express the absence of prior information about model parameters.

We used WinBUGS 1.4.3 to fit the model using Markov chain Monte Carlo (MCMC). MCMC is a simulation tool to draw large samples from the Bayesian posterior distribution of parameters that is typically analytically intractable [13]. The model was executed in R version 3.3.1 using the R_2_WinBUGS library. To enhance convergence of the MCMC sampler, we standardized population density by dividing the difference between each observation and the group mean by their respective standard deviations and also truncated the normal distributions for over dispersion effects to within (−16, 16) by multiplying with an indicator uniform prior distribution [14]. We ran the model for 250,000 iterations and discarded the first 50,000. We checked whether the priors were too restrictive, by inspecting a histogram of the posterior [15], with no such evidence found.

As we were executing two interdependent logit models, the model initially did not appear to find realistic parameter space for incidence rate and case detection rates (with estimated incidence rates far exceeding observed rates). Thus the model was forced into realistic parameter space [16] by restricting the maximum incidence rate to 1%, corresponding to 1000 per 100,000 per year (this is a realistic upper limit, being five times that of current notification rates and only reported in one country in the world in 2015).

Four candidate Bayesian geospatial models were considered:

Model 1 Covariate only; *logit*(*π*_*ij*_) = *β* + *β*_*1*_ *X*_*i*_ + *β*_*2*_ *Z*_*i j*_ + *β*_*3*_ *logit*(*π*_*ij* − *1*_)*.*

Model 2 Covariates and non-spatially correlated random effect; *logit*(*π*_*ij*_) = *β* + *β*_*1*_ *X*_*i*_ + *β*_*2*_ *Z*_*i j*_ + *β*_*3*_ *logit*(*π*_*ij* − *1*_) + *ν*_*ij*_ *.*

Model 3 Covariate and spatially correlated random effect; *logit*(*π*_*ij*_)_=_ *β* + *β*_*1*_ *X*_*i*_ + *β*_*2*_ *Z*_*i j*_ + *β*_*3*_ *logit*(*π*_*ij* − *1*_) + *ɛ*_*i*_ *.*

Model 4 Full Model with covariates and all random effects included *logit*(*π*_*ij*_) = *β* + *β*_*1*_ *X*_*i*_ + *β*_*2*_ *Z*_*i j*_ + *β*_*3*_ *logit*(*π*_*ij* − *1*_) + *ν*_*ij*_ + *ɛ*_*i*_ *.*

Covariates and random effects were included/excluded to determine if this had an effect on model fit and to determine the extent to which they accounted for the spatial correlation. The effect of spatially correlated random effects was assessed by examining the credible intervals (CrI) of the coefficients of the selected covariates and incidence and case detection estimates.

The deviance information criterion (DIC) statistic was calculated for the models with or without random effect terms to determine if the addition of the geospatial component improved model fit. Maps of the summary statistics of the posterior distributions of predicted incidence and the notified cases were constructed using R.

### Goodness-of-fit

We conducted posterior predictive checks to evaluate whether the models considered could likely have generated datasets that are similar to our observed dataset. This procedure uses parameter values estimated by the model using observed data to generate simulated data sets. Chi-square fit statistic was calculated to quantify the lack of fit both for the observed data and for the simulated data sets, and a Bayesian *P*-value was calculated to quantify the ratio between the fit statistic for the observed data and that of the simulated (perfect datasets under model assumptions) (Additional file 1). A Bayesian P-value close to 0.5 indicates a model fits the data, while a P-value close to zero or one suggests poor fit [15, 17, 18].

## Results

### Model selection

We found that a binomial mixture model containing the spatially structured random effect and covariates was the best fitting model, and that the model with no random effects included was the poorest fitting. When a spatially structured random effect was included into the covariate model, the DIC dropped by more than 230 whereas inclusion of the non-spatially correlated random effect reduced the DIC by only 123 (Additional file 2).

### The role of the spatially correlated random effect

As well as the marked reduction in the DIC, the coefficient for the temporal lag in the state-space model became non-significant while the credible intervals surrounding other coefficients widened, but remained significant, after inclusion of the geospatial component in the state-space model. Similarly, the inclusion of non-spatially structured random effects resulted in non-significant temporal lag. By contrast, the credible intervals surrounding the predicted case detection and incidence rates became more precise when the geospatial component was added to the model (Additional file 2). In the covariate only model, the coefficient for the spatial lag covariate was not statistically significant, but became significant with the inclusion of both random effects.

### Incidence and case detection rates

Outputs from the best fitting model are presented in Table 1 and show the estimated TB incidence in Sheka Zone was 198 (95% CrI: 187, 233) per 100,000 per year in 2010 and 232 (95% CrI: 212, 253) per 100,000 per year in 2014.

**Table 1:** Incidence and case detection estimates in Sheka zone, Ethiopia

Year	Median, 95% credible interval	
	Estimated incidence	Case detection rate
2010	198 (187, 233)	60 (49, 72)
2011	218 (199, 238)	65 (53, 77)
2012	216 (200, 234)	59 (47, 71)
2013	219 (203, 236)	67 (56, 77)
2014	232 (212, 253)	71 (60, 81)

**Fig. 1:**
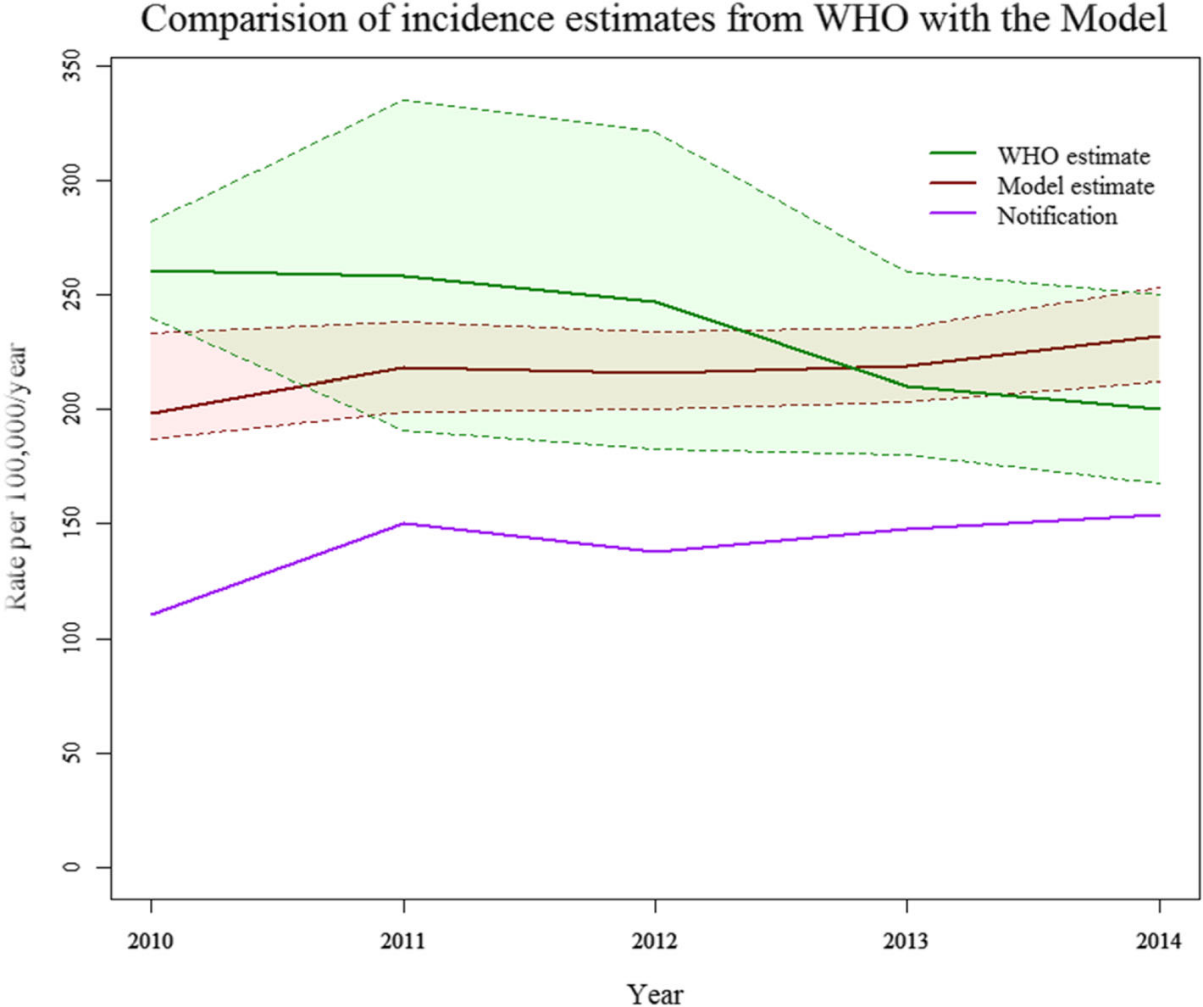
Comparison of WHO estimated TB incidence for Ethiopia, with the model estimated incidence using data from Sheka Zone, Ethiopia (*Broken lines represent the upper and lower 95% credible intervals)*

The model demonstrated a wide discrepancy between the estimated incidence rate and the notification rate, with estimated incidence 1.4 (2014) to 1.7 (2010) times the notification rate, placing CDR at 60% (95% CrI: 49, 72) in 2010 to 71% (95% CrI: 60, 81) in 2014 (Fig. 1).

### Covariate effects on incidence and detectability

Coefficients of the covariates included in the four candidate hierarchical binomial mixture models are presented in the Additional file and the coefficients for the best fitting model are presented in Table 2. Our methodological approach enabled us to present predictors of incidence and CDR separately.

**Table 2:** Predictors of incidence, and case detection rate in the binomial mixture model

	Coefficient, median (95% CrI)	Odds*, Odds ratio (95% CrI)
State-space model		
β_0_ (intercept)	−5.5 (−6.1, −4.9)	0.004 (0.002, 0.007)*
β_1_ (population density)^a^	0.1 (0.1, 0.14)	1.1 (1.1,1.2)
β_2_ (temporal lag)^b^	0.003 (−0.1, 0.1)	1.0 (0.9, 1.1)
β_3_ (spatial lag)^b^	0.5 (0.3, 0.7)	1.6 (1.4, 2.0)
Case detection model		CDR, median (95% CrI)
CDR in *kebeles* with no health facility		0.59 (0.55, 0.63)
CDR in *kebeles* with a health facility		0.69 (0.62, 0.77)

^a^ *Population per square km*, ^b^: *number of incident TB cases/100,000 population/year*

*CDR- case detection rate*

As shown in Table 2, TB incidence rate was positively associated with population density and spatial lag in the geographically adjacent sites. An increase of 10/100,000/year in average TB incidence in adjacent *kebeles* predicted a 5.0/100,000/year increment in TB incidence in an index *kebele.* Similarly, an increase of population size by 10 per square kilometre predicted an increase in TB incidence by 1/100,000/year. Population density remained a significant predictor of TB incidence in all candidate models (Additional file 2). All the models except the covariate only model demonstrated the statistically significant effect of incidence at neighbouring locations. In the best fitting model, TB incidence was not significantly related to incidence at a temporal lag of one year (Table 2).

On the other hand, CDR was related to the presence of a health facility in the best fitting model, as well as in all other candidate models considered in this study. The estimated case detection rate in *kebeles* with no health facility was 59% (95% CrI: 55, 63), while the rate was 69% (95% CrI: 62, 77) in *kebeles* with a health facility.

### Spatial distribution

**Fig. 2:**
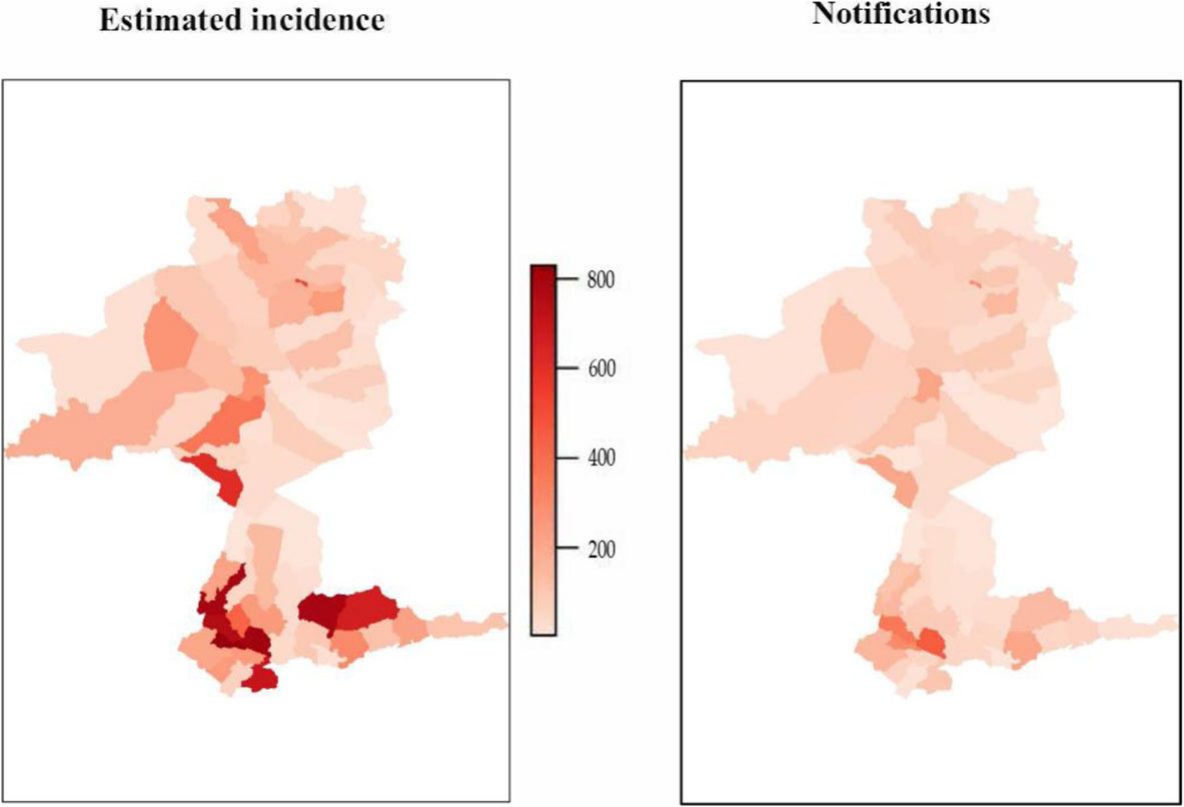
Comparison of spatial distribution of model estimated TB incidence and notifications per 1,000,000, Sheka Zone, Ethiopia, 2010–2014

Maps of the spatial distribution of estimated incidence and notification rates of TB presented in Fig. 2 revealed the presence of undetected TB cases at *kebele* level in Sheka Zone. The patterns observed in the maps of incidence and notifications appeared broadly correlated. However, the incidence map shows areas of considerably greater burden and identifies new, previously unrecognised areas of high burden. The incidence map identified many rural *kebeles* without a health facility and urban *kebeles* as high burden locations, in contrast to the notification map that identified mainly urban *kebeles*. These locations corresponded to *kebeles* with high population density and were surrounded by high incidence *kebeles.* The maps illustrate that estimated incidence rates are higher than notification rates, highlighting that notification data markedly underestimate incidence.

### Goodness of fit of the model

**Fig. 3:**
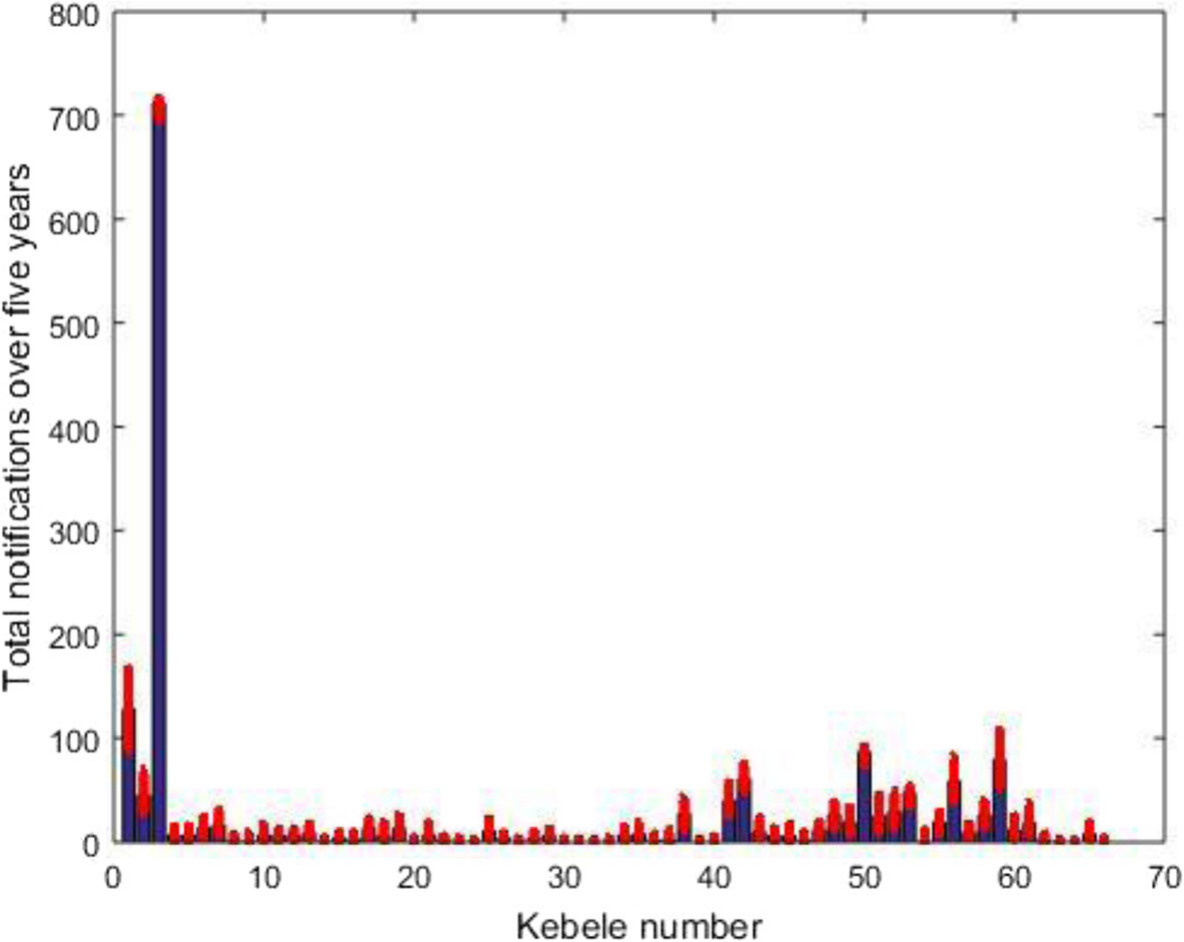
Comparison of the observed (notifications) (in *blue*) and the simulated notifications (in *red*). All the chains in the simulated data are plotted as dots

**Fig. 4:**
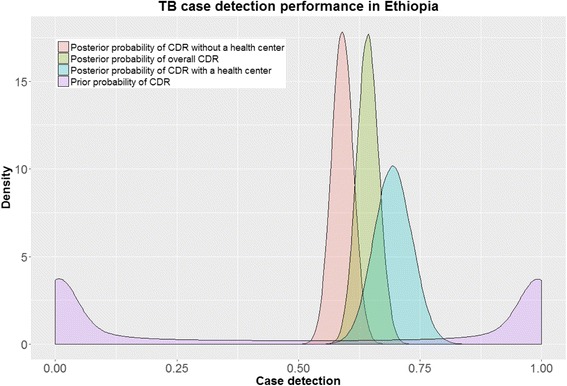
Comparison of prior and posterior probabilities of case detection rate (CDR)

We calculated a Bayesian *P*-value by comparing a Pearson chi-square for both the simulated datasets and the actual dataset. The proportion of times the discrepancy measure for simulated data is greater than the actual data (a Bayesian P-value) from a posterior predictive check was 0.40. The Bayesian P-value suggests that the model fits the observed data satisfactorily [15, 18]. As an internal validity check, we simulated using posterior parameter values whether our model could reproduce the notification data that were previously used to estimate the model parameters. Comparison of notification data with simulated data indicated a close fit (Fig. 3). In addition, comparison of priors with their posteriors also indicated that our estimates were entirely data-driven (Fig. 4).

## Discussion

The Bayesian hidden Markov model approach used in this study provided a means to estimate both TB incidence and case detection rate, and identified previously unrecognised TB hotspots in rural Ethiopia. To our knowledge, this is the first report applying spatially explicit binomial mixture methods to estimate TB incidence and case detection using only case notifications distributed over space and time.

By using Bayesian inference, we were able to incorporate the spatial dependence structure, random effects and a hidden state (true TB incidence) into our model. The improved fit achieved by including a geospatial term highlights the importance of accounting for spatial correlation in TB studies [8]. In this study, incorporation of the spatial dependence structure changed estimated relationships between covariates and incidence: some relationships changed from significant to non-significant, while others remained significant but with reduced precision. Like others [19, 20], we conclude that failing to account for spatial effects may lead to inflated regression coefficients and spuriously narrow credible intervals.

In contrast to previous analyses that described patterns in the notified cases (as a proxy for incidence) [7, 8], our model explicitly separated the true incidence (hidden state) and observation process (case detection). A high or increasing notification rate could reflect either an efficient health system rapidly diagnosing all incident cases or a poor health system failing to detect patients quickly enough to gain control of the epidemic. As these two phenomena lie at opposite poles of programmatic TB control and would be associated with profoundly different case detection rates, it is critical to distinguish them objectively and without the need for opinion-based estimates of case detection [21].

Applied to five years of TB surveillance data in Sheka Zone Ethiopia, our model demonstrated a wide discrepancy between the estimated incidence rate and notification rate in areas with no health centres, which is consistent with WHO estimates [22, 23], highlighting the importance of strengthening surveillance systems to reduce missed cases [23, 24]. Unlike the WHO estimates, however, we estimated that the incidence of tuberculosis is increasing, and that increased notification rates reflect both increased detection and increased incidence.

The estimated TB incidence in this study is highly spatially heterogeneous, replicating previous reports [10, 20, 25], and associated with average incidence in the neghbouring *kebeles* (spatial lag) and population density, reflecting an extended duration of contact between individuals as a driver of TB epidemiology in high-burden settings [26, 27]. In contrast, TB incidence at a temporal lag of one year in this study failed to replicate the statistically significant association observed in the previous analysis using GLMs [10].

In our study, health facility availability predicted high TB case detection. Higher notification rates in some of the studied *kebeles* were attributable to both underlying higher incidence and higher case detection in the setting of health facility availability.

In contrast to methods used by WHO to estimate TB incidence, our approach uses only routinely collected surveillance data and does not incorporate costly prevalence surveys and expert opinion regarding CDR, which is criticized for its insensitivity to recent changes and the potential for bias [1]. Expert opinion is a potentially important strategy to use in a Bayesian models where information is lacking, but has a recognised limitations in accurate adjustment of disease estimates [3] and is widely considered the lowest standard of empirical evidence [28]. In addition, it does not exist at fine geographical levels, such as the one we are assessing here, and so is impractical to use in this context. Hence, our approach uses expert opinion only for the purpose of developing a vague prior probability distribution. We aim to improve on expert opinion through the use of the structured hidden Markov Model and so to develop a feasible alternative approach that can be used for regular monitoring of TB incidence and is reactive to recent changes.

Our case detection model assumes that individual TB cases are detected at a fixed rate and independently (conditional on a given incidence). This assumption may not be valid in the case of concerted efforts at contact tracing. However, as contact tracing is not systematically implemented in Ethiopia, the proportion of cases in our study arising from contact tracing would be small and this assumption would be a minor consideration. Moreover, we included a random effect term (ɷ_ij_) in our model to allow for changes in detection rate over place and time. Sparse data were accounted for by including random effects to explain extra-model variability. Further work is in progress to build models that account for over dispersion, which may arise from non-independent detections of individuals.

## Conclusions

By using Binomial mixture models, we were able to investigate different epidemiological questions related to the size, trend and predictors of TB incidence and CDR. Our model demonstrated a wide discrepancy between incidence rate and notification rates, and identified previously unrecognised TB hotspots in rural Ethiopia, but was broadly consistent with official estimates. This approach provides an alternative approach to estimating incidence, entirely independent of the methods involved in current case detection rate estimates and is feasible to perform from routinely collected surveillance data.

## Additional files

Additional file 1: Model code. (DOCX 14 kb)

Additional file 2: Outputs from Candidate Models. (DOCX 21 kb)

## Abbreviations

CDR: Case detection rate
CrI: Credible Interval
DIC: Deviance information criteria
TB: Tuberculosis
