# Supplementary material for: A novel Bayesian geospatial method for estimating tuberculosis incidence reveals many missed TB cases in Ethiopia
Source: BMC Infect Dis. 2017 Oct 2;17:662. doi: 10.1186/s12879-017-2759-0 (PMC5625624; doi:10.1186/s12879-017-2759-0)
Supplement: Supplementary file 1 — Model code. (DOCX 14 kb) [file 12879_2017_2759_MOESM1_ESM.docx]

**Additional file 1: Model code**

model {

**# Priors**

tau.p <- 1/(sd.p *sd.p)

sd.p ~ dunif(0, 5)

beta0 ~ dunif(-10, 10)

beta1 ~ dunif(-10, 10)

beta2 ~ dunif(-10, 10)

beta3 ~ dunif(-10, 10)

theta1 ~ dunif(-15, 15)

ss[1:N] ~ car.normal (adj[], weights[1:sumNumNeigh], num[], tau.s);

tau.s ~ dgamma(0.5, 0.0005);

for (k in 1:sumNumNeigh){

weights[k] <- 1

}

for( i in 1 : N){

for(j in 1 : 5){

v[i, j] ~ dnorm(0, tau.p) I(-10, 10);

beta[i, j] <-beta0

}

}

**# Likelihood**

for ( j in 1:1) {

for( i in 1 : N) {

**# True State**

Incidence[i, j] ~ dpois(lambda[i, j]);

lambda[i, j] <- m[i, j] * Pop[i, j]; *# m is probability of infection between 0 and 1*

logit(m[i, j]) <- beta+ beta1*X[i] + beta3* Z[i, j] + ɛ[ i] +ν[i,j]

#Observation Process

y[i, j] ~ dbin(p[i, j], Incidence[i, j]);

lp[i, j] <- theta + theta_1_*Xhc[i] + ɷ[i, j]

theta0[i, j] ~ dunif(-10, 10)

p[i, j]<- exp(lp[i, j])/(1 + exp(lp[i, j]))

}

}

for ( j in 2:5) {

for( i in 1 : N) {

**# True State**

Incidence[i, j] ~ dpois(lambda[i, j]);

lambda[i, j] <- m[i, j] * Pop[i, j]

logit(m[i, j]) <- beta + beta_1_*X[i] + beta_2_* Z[i, j] + beta_3_*logit(m[i, j-1]) + ɛ[i] +ν[i,j] **#Observation Process**

y[i, j] ~ dbin(p[i, j], Incidence[i, j]);

lp[i, j] <- theta+ theta_1_*Xhc[i] + ɷ[i, j]

p[i, j]<- exp(lp[i, j])/(1 + exp(lp[i, j]))

theta0[i, j] ~ dunif(-10, 10)

}

}

**#Model Goodness of fit**

# Posterior predictive distributions of χ^2^ discrepancy: assess model fit using χ^2^ discrepancy

for ( j in 1:5) {

for( i in 1 : N) {

# Compute χ^2^ statistic for observed data

Eval[i, j] <- p[i, j] * Incidence[i, j]; # Expected notification using model parameters

E[i, j] <- pow((y[i, j] - Eval[i, j]) , 2) / (Eval[i, j] + 0.5);

**# generate replicate (simulated) data and compute χ^2^ statistic for them**

y.new[i, j] ~ dbin(p[i, j], Incidence[i, j]); #generate notification using the model

E.new[i,j] <- pow((y.new[i,j] - eval[i, j]), 2) / (eval[i,j]+ 0.5)

}

}

fit <- sum(E[ , ])

fit.new <- sum(E.new[, ])

bpv <- step(fit.new - fit); # Bayesian p-value

}

",fill = TRUE)

sink()
