# Supplementary material for: A novel Bayesian geospatial method for estimating tuberculosis incidence reveals many missed TB cases in Ethiopia
Source: BMC Infect Dis. 2017 Oct 2;17:662. doi: 10.1186/s12879-017-2759-0 (PMC5625624; doi:10.1186/s12879-017-2759-0)
Supplement: Supplementary file 2 — Outputs from Candidate Models. (DOCX 21 kb) [file 12879_2017_2759_MOESM2_ESM.docx]

**Additional file 2. Outputs from Candidate Models**

**Table 1: Incidence and case detection estimates from candidate models**

| **Year** | **Incidence, mean, 95%CrI** | **Case detection rate, 95%CrI** | | | **DIC** |
| --- | --- | --- | --- | --- | --- |
| **Model 1: Covariate only model (no random effects)** | | | | | 762 |
| 2010 | 246 (202, 301) | 0.508 (0.421, 0.593) |  | |  |
| 2011 | 342 (299, 392) | 0.454 (0.388, 0.527) |  | |  |
| 2012 | 332 (297, 371) | 0.412 (0.388, 0.488) |  | |  |
| 2013 | 332 (297, 372) | 0.519 (0.451, 0.590) |  | |  |
| 2014 | 334 (299, 376) | 0.536(0.465, 0.608) |  | |  |
| **Model 2: Covariate and non-spatially structured random effects model** | | | | | 649 |
| 2010 | 215 (159, 289) | 0.659(0.545, 0.755) | |  |  |
| 2011 | 223 (178, 278) | 0.692(0.559, 0.794) | |  |  |
| 2012 | 218 (176, 269) | 0.629(0.505, 0.753) | |  |  |
| 2013 | 226 (187, 275) | 0.685(0.591, 0.769) | |  |  |
| 2014 | 261 (219, 312) | 0.714(0.624, 0.786) | |  |  |
| **Model 3: Covariate and spatially structured random effects model** | | | | | 526 |
| 2010 | 198 (187, 233) | 0.606 (0.486, 0.717) |  | |  |
| 2011 | 218 (199, 238) | 0.646 (0.528, 0.767) | | |  |
| 2012 | 216 (200, 234) | 0.587 (0.470, 0.707) | | |  |
| 2013 | 219 (203, 236) | 0.669 (0.559, 0.770) | | |  |
| 2014 | 232 (212, 253) | 0.708 (0.596, 0.806) | | |  |
| **Model 4: Full Model (both random effects in)** | | | | | 657 |
| 2010 | 215 (160, 289) | 0.659(0.534, 0.758) | | |  |
| 2011 | 223 (178, 278) | 0.692(0.563, 0.796) | | |  |
| 2012 | 218 (176, 268) | 0.638(0.509, 0.756) | | |  |
| 2013 | 226 (187, 275) | 0.689(0.592, 0.772) | | |  |
| 2014 | 260 (219, 311) | 0.712(0.619, 0.786) | | |  |

**Table 2: Regression coefficients and predicted case detection rates from the candidate models**

|  | Coefficient, median  (95% credible interval) | | **DIC** | |
| --- | --- | --- | --- | --- |
| **Model 1: Covariate only model (no random effects)** 762 | | | | |
| β_0_ | | -5.25 (-5.54, -4.89) |  |  |
| β_1_ (pop density) | | 0.093 (0.076, 0.113) |  |  |
| β_2_ (temporal lag) | | -0.081 (-0.142, -0.020) |  |  |
| β_3_ (spatial lag) | | 0.085 (-0.103, 0.357) |  |  |
| CDR without a HC | | 0.40 (0.36, 0.44) |  |  |
| CDR with a HC | | 0.57 (0.51, 0.64) |  |  |
| **Model 2: Covariate and non-spatially structured random effects** 649 | | | | |
| β_0_ | | -5.86 (-6.46, -5.25) |  |  |
| β_1_ (pop density) | | 0.064 (0.038, 0.092) |  |  |
| β_2_ (temporal lag) | | 0.037 (-0.055, 0.129) |  |  |
| β_3_ (spatial lag) | | 0.462 (0.172, 0.761) |  |  |
| CDR without a HC | | 0.54 (0.49, 0.59) |  |  |
| CDR with a HC | | 0.80 (0.69, 0.92) |  |  |
| **Model 3: Covariate and spatially structured random effects** 526 | | | | |
| β_0_ | | -5.49 (-6.06, -4.92) |  |  |
| β_1_(pop density) | | 0.109 (0.083, 0.141) |  |  |
| β_2_(temporal lag) | | 0.0026 (-0.050, 0.054) |  |  |
| β_3_(spatial lag) | | 0.478 (0.306, 0.674) |  |  |
| CDR without a HC | | 0.59(0.55, 0.63) |  |  |
| CDR with a HC | | 0.69(0.62, 0.77) |  |  |
| **Model 4: Full Model (both random effects included)** 657 | | | | |
| β_0_ | | -5.8 (-6.5, -5.2) |  |  |
| β_1_ (pop density) | | 0.065 (0.038, 0.095) |  |  |
| β_2_ (temporal lag) | | 0.038 (-0.056, 0.13) |  |  |
| β_3_ (spatial lag) | | 0.46 (0.17, 0.78) |  |  |
| CDR without a HC | | 0.54 (0.49, 0.59) |  |  |
| CDR with a HC | | 0.80 (0.68, 0.93) |  |  |
|  | |  |  |  |

*CDR-case detection rate, HC- health centre*
